# Transdiagnostic treatment of depression and anxiety: a meta-analysis

**DOI:** 10.1017/S0033291722003841

**Published:** 2023-10

**Authors:** Pim Cuijpers, Clara Miguel, Marketa Ciharova, David Ebert, Mathias Harrer, Eirini Karyotaki

**Affiliations:** 1 Department of Clinical, Neuro and Developmental Psychology, Amsterdam Public Health Research Institute, Vrije Universiteit Amsterdam, Amsterdam, the Netherlands; 2 WHO Collaborating Centre for Research and Dissemination of Psychological Interventions, Vrije Universiteit Amsterdam, Amsterdam, the Netherlands; 3 Babeș-Bolyai University, International Institute for Psychotherapy, Cluj-Napoca, Romania; 4 Psychology & Digital Mental Health Care, Technical University Munich, Munich, Germany; 5 Clinical Psychology & Psychotherapy, Friedrich-Alexander University Erlangen-Nuremberg, Erlangen, Germany

**Keywords:** Anxiety, depression, meta-analysis, psychotherapy, transdiagnostic treatment

## Abstract

**Background:**

In the past 10 years an increasing number of randomised trials have examined the effects of transdiagnostic treatments of patients with depression or anxiety. We conducted the first comprehensive meta-analysis of the outcomes of this emerging field.

**Methods:**

We used the searches in PubMed, PsychINFO, Embase and the Cochrane library of an existing database of randomised trials of psychological interventions for depression to identify studies comparing a transdiagnostic treatment of patients with depression or anxiety with a control group (deadline 1 January 2022). We conducted random-effects meta-analyses and examined the effects on depression and anxiety at the short and longer term.

**Results:**

We included 45 randomised controlled trials with 51 comparisons between a psychotherapy and a control group and 5530 participants. Thirty-five (78%) studies were conducted in the last 10 years. The overall effect size was g = 0.54 (95% CI 0.40–0.69; NNT = 5.87), with high heterogeneity (*I*^2^ = 78; 95% CI 71–83), and a broad PI (−0.31–1.39). The effects remained significant in a series of sensitivity analyses, including exclusion of outliers, adjustment for publication bias, for studies with low risk of bias, and in multilevel analyses. The results were comparable for depression and anxiety separately. At 6 months after randomisation the main effects were still significant, but not at 12 months, although the number of studies was small.

**Conclusions:**

Transdiagnostic treatments of patients with depression or anxiety are increasingly examined and are probably effective at the short term.

## Introduction

Depression and anxiety disorders are highly prevalent (Santomauro et al., 2022; World Health Organization, 2022), are associated with considerable loss of quality of life for patients and their relatives, and with an enormous disease burden and economic costs at the population level (Whiteford, Ferrari, Degenhardt, Feigin, & Vos, 2015). Several psychological interventions have been developed for the treatment of depression and anxiety disorders. Most of these treatments focus on one specific disorder and the large majority of randomised trials examining the effects of these treatments have also focused on one specific disorder (Cuijpers, Cristea, Karyotaki, Reijnders, & Huibers, 2016). However, comorbidity between depression and anxiety has been estimated to be as high as 60% for case-level disorders (Kessler et al., 2011, 2015) and probably even higher when subthreshold cases are included. Mixed anxiety and depression has also been found to be more common than ‘pure diagnoses’ in the community and is a frequent presentation in primary care (Newby, Mewton, Williams, & Andrews, 2014). It could be considered therefore that depression and anxiety in fact constitute one cluster of neurotic disorders and share similar psychological and biological mechanisms (Tyrer, Tyrer, & Guo, 2016). Furthermore, psychological treatments of depression and anxiety often share the same core elements, especially in cognitive behavioural interventions. In addition, several pharmacological treatments have also been found to be effective in both depression and anxiety disorders (Cipriani et al., 2018; Gosmann et al., 2021).

It should not come as a surprise therefore that a growing number of studies have examined the effects of treatments that focus on both depression and anxiety, especially in the past 10 years. These ‘transdiagnostic’ treatments include components that have been found to be effective in the treatment of both depression and anxiety, such as cognitive restructuring (Ciharova et al., 2021), but also focus on components that are specifically aimed at depression, such as behavioural activation (Ciharova et al., 2021; Cuijpers et al., 2021), or anxiety, such as exposure (Freitas et al., 2021; Ougrin, 2011). This makes it possible that patients with depression only, with anxiety only or with both can benefit from such interventions. Such transdiagnostic treatments have the advantage that they focus more on comorbidity compared to disorder-specific interventions and can build on the similar aetiological and maintenance processes underlying depressive and anxious psychopathology. There is growing consensus in the field that a novel approach is needed in the way we classify, formulate, treat, and prevent depression and anxiety disorders (Newby, McKinnon, Kuyken, Gilbody, & Dalgleish, 2015). Transdiagnostic approaches to depression and anxiety focus on identifying common and core maladaptive temperamental, psychological, cognitive, emotional, interpersonal and behavioural processes that can be targeted in treatment.

Several meta-analyses have examined the effects of transdiagnostic treatments of depression and anxiety disorders. However, many of these meta-analyses focus only on small subsets of studies, like internet-based transdiagnostic treatments (Newby, Twomey, Li, & Andrews, 2016; Păsărelu, Andersson, Nordgren, & Dobrean, 2017), interventions using the Unified protocol for transdiagnostic interventions (Sakiris & Berle, 2019), acceptance and commitment therapy (Thompson, Destree, Albertella, & Fontenelle, 2021), compare transdiagnostic with diagnosis-specific treatments (Pearl & Norton, 2017), or focus on transdiagnostic treatments of anxiety disorders, while excluding studies in depression (Reinholt & Krogh, 2014). One meta-analysis focusing on depression or anxiety disorders either included only a small sample of studies overall, with hardly any study in patients with depression or anxiety (Andersen et al., 2016). One more meta-analysis included a larger sample of trials but the majority of the studies only focused on anxiety disorders and only a minority (15) included both patients with depression as well as anxiety disorders (Newby et al., 2015). The actual number of trials on depression or anxiety is, however, three times as large (see below).

One problem of meta-analyses of transdiagnostic treatments is that the searches are susceptible for missing relevant trials, because there are no clear search terms for transdiagnostic treatments. That means that searches either have to be very broad (with considerable work for the meta-analysts) or more narrow, with a larger risk of missing studies. In the database we have developed for psychological treatments of depression (Cuijpers & Karyotaki, 2020), we have done these very broad searches, which also identify transdiagnostic treatments (as long as they include depression). We used these searches in the current meta-analysis to identify relevant studies on transdiagnostic treatments, which are broader than the searches of previous meta-analyses (more than 30 000 records, compared to 10 000 in the largest previous meta-analysis on transdiagnostic therapies; Newby et al., 2015). This means that because of our broad searches, we can be more confident that we have included all relevant studies on transdiagnostic treatments. The goal of the current meta-analysis is therefore to extend previous meta-analyses with more comprehensive searches and include trials that have not been included in previous meta-analyses, resulting in a more comprehensive overview of the literature.

In the current paper, we examined the effects of transdiagnostic treatments for patients with depression or anxiety, compared to control conditions. We conducted a meta-analysis with a broader search than has been possible in previous meta-analyses, in the hope that we can provide the most comprehensive estimate of these treatments up to now. In addition to the main analyses, we also conducted subgroup analyses to examine potential differences between subsets of studies and sources of heterogeneity. Because we expected a relatively small number of trials and power is generally low for subgroup analyses, we limited these analyses to a small set of essential variables.

## Methods

### Identification and selection of studies

The current study is part of a larger meta-analytic project on psychological treatments of depression that was registered at the Open Science Framework (Cuijpers & Karyotaki, 2020; doi:10.17605/OSF.IO/825C6) and online supplemental materials are available at the website of the project (www.metapsy.org). This database has been used in a series of earlier published meta-analyses (Cuijpers, 2017). The protocol for the current meta-analysis has been published at the Open Science Framework (Cuijpers et al., 2022a, 2022b; https://osf.io/kyga2).

The studies included in the current study were identified through the larger, already existing database of randomised trials on the psychological treatment of depression. For this database we searched four major bibliographical databases (PubMed, PsycINFO, Embase and the Cochrane Library) by combining index and free terms indicative of depression and psychotherapies, with filters for randomised controlled trials. The full search strings can be found at the website of the project (www.metapsy.org) and are given in Appendix A. Furthermore, we checked the references of earlier meta-analyses on psychological treatments of depression. The database is continuously updated and was developed through a comprehensive literature search (from 1966 to 1 January 2022). All records were screened by two independent researchers and all papers that could possibly meet inclusion criteria according to one of the researchers were retrieved as full-text. The decision to include or exclude a study in the database was also done by the two independent researchers, and disagreements were resolved through discussion.

Because the searches for this larger database are aimed at identifying trials on psychotherapies for depression, they automatically also identify trials in people with depression or anxiety (because these trials can be assumed to include depression as search term). In the current meta-analysis, we specifically focus on trials of these transdiagnostic treatments of patients with depression or anxiety. Next to the searches in the bibliographic databases, we also checked the references of earlier meta-analyses of transdiagnostic treatments of depression and anxiety (Andersen et al., 2016; Newby et al., 2015, 2016; Păsărelu et al., 2017; Pearl & Norton, 2017; Reinholt & Krogh, 2014; Sakiris & Berle, 2019; Thompson et al., 2021).

We included randomised controlled trials in which a psychological intervention for people with depression or anxiety is compared with a control condition (waitlist, care-as-usual, other) in adults (older than 18 years). Trials had to examine the effects of the intervention in a mixed sample that included participants with depression, anxiety, or comorbid anxiety and depression. Depression and anxiety can be defined as meeting criteria for a depressive or anxiety disorder according to a diagnostic interview or as a score above the cut-off on a self-report depression measure. Studies in which all participants had a depressive disorder were excluded, because they are already included in our previous meta-analyses on psychotherapies for depression (Cuijpers et al., 2021). Similarly, we excluded studies that were focused on participants exclusively diagnosed with anxiety disorders. For diagnosed anxiety disorders we focused on panic disorder, social anxiety disorder and generalised anxiety disorder, as indicated in the DSM5. We only included individual, group and guided self-help interventions. Interventions without any human interaction (unguided self-help) were not included, because these have been found to be significantly less effective than other treatment formats (Cuijpers, Noma, Karyotaki, Cipriani, & Furukawa, 2019; Karyotaki et al., 2017, 2021). We did not exclude studies based on setting, type of therapy or specific characteristics of participants. We did exclude studies in which two therapies were compared with each other and no control group was available.

### Quality assessment and data extraction

We assessed the validity of included studies using four criteria of the ‘Risk of bias’ (RoB) assessment tool, version 1, developed by the Cochrane Collaboration (Higgins et al., 2011). We used version 1 of this tool because this meta-analysis is included in the broader meta-analytic project of psychological treatments of depression, and therefore this meta-analysis builds on previously conducted RoB assessments (Sterne et al., 2019).

The RoB tool assesses possible sources of bias in randomised trials, including the adequate generation of allocation sequence; the concealment of allocation to conditions; the prevention of knowledge of the allocated intervention (masking of assessors); and dealing with incomplete outcome data (this was assessed as positive when intention-to-treat analyses were conducted, meaning that all randomised patients were included in the analyses). Assessment of the validity of the included studies was conducted by two independent researchers, and disagreements were solved through discussion.

We also coded participant characteristics (diagnostic method; recruitment method; target group; mean age; proportion of women); characteristics of the psychological treatments (type of therapy; treatment format; number of sessions) as well as general characteristics of the studies (type of control group; publication year; country where the study was conducted). The details of these characteristics can be found at the website of the project (www.metapsy.org).

### Outcome measures

For each comparison between a psychological treatment and a control condition, the effect size indicating the difference between the two groups at post-test was calculated (Hedges' g; Hedges & Olkin, 1985). Effect sizes were calculated by subtracting (at post-test) the average score of the psychotherapy group from the average score of the control group and dividing the result by the pooled standard deviation. Because some studies were expected to have relatively small sample sizes we corrected the effect size for small sample bias. When means and standard deviations were not reported we calculated the effect size using dichotomous outcomes or change scores; and if these were not available either, we used other statistics (such as *t*-value or *p* value) to calculate the effect size.

For each study we calculated the effect size indicating the effects of the intervention on depression and anxiety. We examined the effects of the pooled outcomes on depression and anxiety together, but also separately for depression and for anxiety.

### Meta-analyses

Analyses were conducted using the ‘metapsyTools’ (Harrer, Kuper, & Cuijpers, 2022) package in R (version 4.1.1) and RStudio (version 1.1.463 for Mac). The metapsyTools package was specifically developed for the meta-analytic project of which this study is part of. The package imports functionality of the ‘meta’ (Balduzzi, Rücker, & Schwarzer, 2019), ‘metafor’ (Viechtbauer, 2010), and ‘dmetar’ (Harrer, Cuijpers, Furukawa, & Ebert, 2021) packages.

We calculated the pooled effect sizes in several different ways, as implemented in the metapsyTools package, so that we could explore if different pooling methods result in different outcomes. In our main analysis model, all effect size data that was available for a comparison in a specific study was aggregated within that comparison first. These aggregated effects were then pooled across studies and comparisons. To aggregate effects within comparisons, an intra-study correlation coefficient of *ρ* = 0.5 was assumed.

We conducted several other analyses to examine whether these main outcomes were robust. First, we used the same method as in the main model, but performed the first aggregation step on a study level (*v.* on a comparison level; i.e. multiple treatment arms within a study were pooled within the study). Second, we estimated the overall effect using a hierarchical three-level meta-analytic model (effect sizes nested in studies), applying robust variance estimation (RVE) to ensure that estimates are approximately unbiased even when the model itself is not perfectly specified (Hedges, Tipton, & Johnson, 2010). Third, we estimated the pooled effect using a three-level ‘correlated and hierarchical effects’ (CHE) model, which was recently proposed by Pustejovsky and Tipton (2022); parameter tests and confidence intervals of which were also calculated using RVE to guard against model misspecification. We assumed an intra-study correlation of *ρ* = 0.5 for this model. Fourth, we calculated the effect when only the smallest or largest effect in each study was considered. Fifth, we pooled effects while excluding outliers, using the ‘non-overlapping confidence intervals’ approach, in which a study is defined as an outlier when the 95% CI of the effect size does not overlap with the 95% CI of the pooled effect size (Harrer et al., 2021). Sixth, we pooled effects while excluding influential cases as defined by the diagnostics in Viechtbauer and Cheung (2010). Seventh, we adjusted the pooled effect size for publication bias, using Duval and Tweedie's trim and fill procedure (Duval & Tweedie, 2000), which yields an estimate of the effect size after correction for the funnel plot asymmetry. Eighth, we estimated the pooled effect using only studies with low risk of bias. Lastly, as another sensitivity analysis, we calculated the pooled effect size under the assumption that all effect sizes were independent.

A random-effects model was assumed for all analyses. Between-study heterogeneity variances (components) were estimated using restricted maximum likelihood. For models not fitted using RVE, we applied the Knapp-Hartung method to obtain robust confidence intervals and significance tests of the overall effect (IntHout, Ioannidis, & Borm, 2014). As a test of homogeneity of effect sizes, we calculated the *I^2^*-statistic and its 95% confidence interval, which is an indicator of heterogeneity in percentages. A value of 0% indicates no observed heterogeneity, and larger values indicate increasing heterogeneity, with 25% as low, 50% as moderate, and 75% as high heterogeneity (Higgins, Thompson, Deeks, & Altman, 2003). For the three-level models, we calculated a multilevel extension of *I^2^*, which describes the amount of total variability attributable to heterogeneity within studies (level 2) and heterogeneity between studies (level 3) (Cheung, 2014; Harrer et al., 2021). Because *I*^2^ cannot be interpreted as an absolute measure of the between-study heterogeneity, we also added the prediction interval (PI), which indicates the range in which the true effect size of 95% of all populations will fall (Borenstein, Hedges, Higgins, & Rothstein, 2009; Borenstein, Higgins, Hedges, & Rothstein, 2017).

In addition to Hedges' g, we also calculated the Numbers-needed-to-treat (NNT) using the formulae provided by Furukawa (1999), in which the control group's event rate was set at a conservative 17% (based on the pooled response rate of 50% reduction of symptoms across trials in psychotherapy for depression) (Cuijpers et al., 2021).

We conducted a series of pre-planned subgroup analyses, examining the effects of the interventions according to type of treatment, treatment format, target group, recruitment method and type of control group. We avoided subgroups with less than three studies.

We also conducted two post-hoc subgroup analyses, one on year of publication (in three categories), and one on studies that included a small sample of patients with obsessive-compulsive disorder (OCD: <10%) compared to studies that did not include such patients.

Apart from calculating the effect size after adjustment for publication bias using Duval and Tweedie's trim and fill procedure, we tested for publication bias by inspecting the funnel plot on primary outcome measures and by Egger's test of the intercept to quantify the bias captured by the funnel plot and to test whether it was significant.

## Results

### Selection and inclusion of studies

**Fig. 1.**
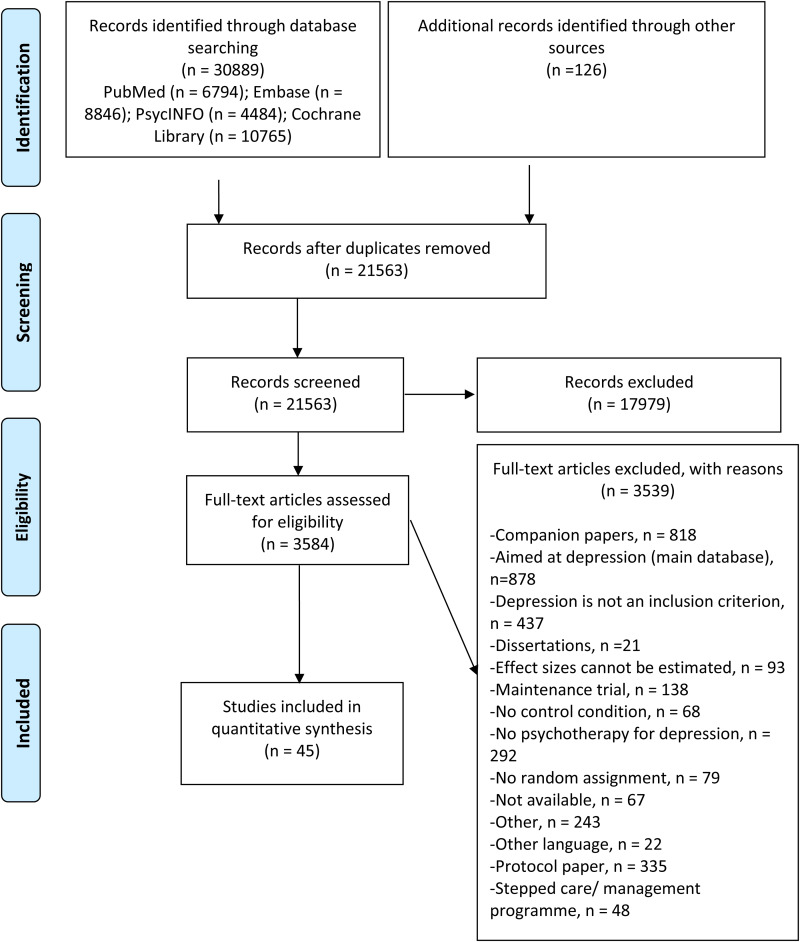
PRISMA flowchart for the inclusion of studies.

After examining a total of 30 889 records (21 563 after removal of duplicates), we retrieved 3584 full-text papers for further consideration. We excluded 3537 of the retrieved papers. The PRISMA flowchart describing the inclusion process, including the reasons for exclusion, is presented in Fig. 1. A total of 45 randomised controlled trials (with 51 comparisons between a psychotherapy and a control group) met inclusion criteria for this meta-analysis.

### Characteristics of included studies

A summary of key characteristics of the 45 included studies is presented in Table 1. References are given in Appendix B. In the trials, 5530 patients participated, 2964 in the intervention and 2566 in the control conditions. In 14 trials participants met criteria for a depressive or anxiety disorder according to a diagnostic interview, while the other 31 trials included participants who scored above a cut-off on a self-report depression or anxiety scale. In the 14 trials in which participants had to meet diagnostic criteria for a depressive or anxiety disorder, 5 indicated that any depressive or anxiety disorder was included (without specification), one included only major depressive disorder (MDD) and generalised anxiety disorder (GAD), one only MDD and panic disorder, and the others included multiple specified disorders. There were three studies that included a small group of patients with OCD (<10%; Díaz-García *et al*. 2021; González-Robles *et al*. 2020; Kladnitski *et al*. 2020). We included these because in the DSM-IV, OCD was considered to be an anxiety disorder, but we also conducted a subgroup analysis in which we compared the studies in which OCD was included with the other studies.

**Table 1.** Selected characteristics of included studies

Study^a^	recr	diagn	M age	Prop women	Target group	Ther	Form	N sess	Ctr	Coun-try	sg	ac	ba	itt	RoB
Ali (2003)	oth	cut-off	nr	1.00	Lower middle class women	other	ind	8	cau	oth	+	−	sr	0	2
Bathgate (2021)	oth	cut-off	32	0.81	Gen med (cystic fibrosis)	cbt	Other	6	cau	us	+	−	sr	0	2
Calleo (2015)	oth	diag	63	0.12	Gen med (Parkinson)	cbt	Other	8	cau	us	+	+	+	0	3
(Compen, 2018 ftf)	com	cut-off	52	0.86	Gen med (cancer)	3rd	grp	8	cau	eu	+	+	+	+	4
						3rd	gsh	8							
Dao (2011)	oth	cut-off	64	0.22	Gen med (coronary)	cbt	ind	4	cau	us	+	−	sr	+	4
Den Boer (2007)	clin	diag	41	0.66	Adults in general	cbt	Other	10	cau	eu	+	+	sr	+	4
Díaz-García et al. (2021)	com	diag	34	0.72	Adults in general	tibp	gsh	12	wl	eu	+	+	sr	+	4
						tibp + pa	gsh	16	wl	eu	+	+	sr	+	4
Doyle (2017)	com	cut-off	68	0.65	Gen med (obstruct pulmon.)	cbt	Other	8	other	au	+	+	sr	+	4
Ezegbe (2019)	oth	cut-off	21	0.64	College students	cbt	grp	20	wl	oth	+	−	sr	+	3
Fernandez (2020)	oth	cut-off	51	0.94	Gen med (cancer)	3rd	grp	12	wl	eu	+	−	sr	−	1
						bat	grp	12	wl	eu	+	−	sr	−	1
González-Robles et al. (2020)	clin	diag	38	0.69	Adults in general	cbt	gsh	12	cau	eu	+	+	sr	+	4
Gunnarson (2018)	oth	diag	42	0.83	Adults in general	other	ind	5	cau	eu	+	+	+	−	3
Hamilton (2020)	oth	cut-off	31	1.00	Perinatal women	dyn	ind	16	cau	au	−	−	sr	−	1
Heller (2020)	com	cut-off	32	1.00	Perinatal women	pst	gsh	5	cau	eu	+	+	sr	+	4
Hynninen (2010)	com	cut-off	61	0.51	Gen med (COPD)	cbt	grp	7	cau	eu	−	+	sr	+	3
Johansson (2013)	com	diag	45	0.82	Adults in general	dyn	gsh	8	other	eu	+	+	sr	+	4
Kladnitski et al. (2020)	com	diag	39	0.86	Adults in general	cbt	gsh	6	cau	au	+	+	sr	+	4
						3rd	gsh	6	cau	au	+	+	sr	+	4
						other	gsh	6	cau	au	+	+	sr	+	4
Kunik (2008)	com	cut-off	66	0.04	Gen med (COPD)	cbt	grp	8	other	us	+	−	sr	+	3
Lam (2010)	clin	cut-off	72	0.59	Older adults	pst	ind	3	other	eas	+	+	sr	+	4
Lerma (2017)	oth	cut-off	42	0.53	Gen med (haemodialysis)	cbt	grp	5	wl	oth	−	−	sr	−	1
Lindegaard (2020)	com	cut-off	38	0.66	Arabic speaking	cbt	gsh	7	wl	eu	+	−	sr	+	3
Lo (2013)	com	cut-off	44	0.73	Adults in general	3rd	grp	8	wl	eas	+	+	sr	+	4
Mahmoodi (2021)	clin	diag	27	0.53	Comorbid perfectionism	Cbt-pf	ind	12	wl	oth	+	−	sr	−	2
						Cbt-up	ind	12	wl	oth	+	−	sr	−	2
Mead (2005)	clin	cut-off	39	0.68	Adults in general	cbt	gsh	4	wl	uk	+	+	sr	−	3
Mullin (2015)	com	cut-off	28	0.64	College students	cbt	gsh	5	wl	au	+	−	sr	+	3
Muntingh (2016)	clin	diag	47	0.66	Adults in general	other	ind	13	cau	eu	+	+	sr	+	4
Newby (2013)	com	cut-off	44	0.78	Adults in general	cbt	gsh	6	wl	uk	+	+	sr	+	4
Nissen (2020)	oth	cut-off	55	0.91	Gen med (cancer)	3rd	gsh	8	wl	eu	+	+	sr	+	4
Norlund (2018)	oth	cut-off	60	0.33	Gen med (myocard. infarct.)	cbt	gsh	10	cau	eu	+	+	sr	+	4
Olason (2018)	oth	cut-off	36	0.65	Gen med (chronic pain)	cbt	ind	12	cau	eu	−	−	sr	+	2
Pachankis (2020)	com	cut-off	26	nr	Minority women	cbt	ind	10	wl	us	+	−	sr	+	3
Patel (2003)	clin	cut-off	48	0.81	Adults in general	other	ind	6	other	oth	+	−	+	+	3
Ponsford (2016)	clin	cut-off	42	0.27	Gen med (traum. brain inj.)	cbt	ind	15	wl	au	+	+	sr	+	4
Proudfoot (2004)	clin	cut-off	44	0.74	Adults in general	cbt	gsh	7	cau	uk	+	+	sr	+	4
Ren (2019)	oth	cut-off	47	1.00	Gen med (breast cancer)	cbt	grp	9	cau	eas	−	+	+	+	3
Richards (2020)	clin	cut-off	29	0.71	Adults in general	cbt	gsh	6	wl	uk	+	+	sr	+	4
Scheidt (2013)	oth	diag	49	1.00	Gen med (fibromyalgia)	dyn	ind	25	cau	eu	−	+	sr	+	3
Schneider (2021)	com	cut-off	58	0.59	Gen med (acute coronary)	cbt	gsh	5	wl	can	+	+	sr	+	4
Titov (2011)	com	diag	44	0.73	Adults in general	cbt	gsh	8	wl	au	+	+	sr	+	4
Torres (2019)	clin	cut-off	68	0.72	Older adults	3rd	grp	8	cau	can	+	+	sr	−	3
Trimmer (2018)	clin	diag	43	0.54	Adults in general	cbt	grp	9	cau	can	+	−	sr	−	3
Troeung (2014)	com	diag	66	0.33	Gen med (Parkinson)	cbt	grp	8	wl	au	+	+	sr	+	4
Van Beek (2013)	oth	diag	49	0.43	Gen med (noncard. chest pain)	cbt	ind	6	cau	eu	+	+	+	+	4
Wuthrich (2019)	oth	cut-off	69	0.36	Gen med (Parkinson)	cbt	Other	10	wl	au	+	+	sr	−	3
Yorke (2016)	oth	cut-off	47	0.57	Gen med (asthma)	cbt	grp	8	cau	uk	+	+	sr	−	3

3^rd^, third wave therapy; Ac, allocation concealment; Au, Australia; Ba, blinded assessment; Bat, behavioural activation therapy; Can, Canada; Cau, care-as-usual; Cbt-pf, cbt for perfectionism; Cbt-up, unified cbt protocol; cbt, cognitive behaviour therapy; Clin, clinical; Ctr, control; Diag, diagnosed disorder according to a clinical interview; Diagn, diagnosis; Dyn, psychodynamic therapy; Eas, East Asia; Eu: Europe; Grp, group; Gsh, guided self-help; Ind, individual; Itt, intention to treat; M age, mean age; myocard. Infarct, myocaridal infarction; N sess, number of sessions; noncard. chest pain, noncardiac chest pain; Nr, not reported; obstruct pulmon, chronic obstructive pulmonary disease; Oth, other; Pst, problem-solving therapy; Recr, recruitment; RoB, risk of bias tot al score; Sg, sequence generation; Sr, self-report; ther, therapy; Tibp, transdiagnostic internet-based protocol; Tibp + pa, transdiagnostic internet-based protocol with positive affect component; traum. brain inj, traumatic brain injury; Uk, United Kingdom; Us, United States of America; wl, waitlist. ^a^These are the references of the studies included in the meta-analysis and they are given in the Supplemental materials.

In 16 studies, participants were recruited through the community, 12 through clinical referrals and 17 used other recruitment methods. Fifteen studies were aimed at adults in general, 20 on people with general medical disorders (the specific disorders are reported in Table 1), 2 at older adults, 2 at women with perinatal depression, and 5 were aimed at other specific target groups. In 24 studies, usual care was used as control group, 18 studies used a waitlist control group and the 5 remaining studies used another control group (including for example social active control, clinical monitoring, health education and pill placebo). Twenty-one studies were conducted in Europe, 8 in North America, 8 in Australia, and 8 in other countries. All studies were published after 2003, with 8 studies published up to 2010, 10 between 2011 and 2015, and the remaining 27 studies after 2015 (13 in 2020 and 2021). A total of 78% of trials was conducted in the past 10 years.

The 45 trials included 51 interventions arms that were compared with a control group. Thirty-one of the intervention arms examined CBT, 7 third-wave therapies, 3 psychodynamic therapies, and 10 other therapies. Fourteen interventions had an individual format, 13 had a group format, 19 a guided self-help format and the remaining 5 studies had a mixed format. The number of sessions ranged from 3 to 25, with the majority (37 interventions) between 6 and 12 sessions.

Thirty-nine of the 45 studies reported an adequate sequence generation (86.7%); 30 reported allocation to conditions by an independent party (66.7%); 6 reported using blinded outcome assessors (13,3%) while 39 used only self-report outcomes (86.7%). In 32 studies, intent-to-treat analyses were conducted (71.1%). Twenty-two studies (48,9%) met all criteria for low risk of bias, 20 studies (44,4%) met 2 or 3 criteria, and 3 met only one criterion (6,7%).

### Effects of psychological interventions on depression and anxiety

**Fig. 2.**
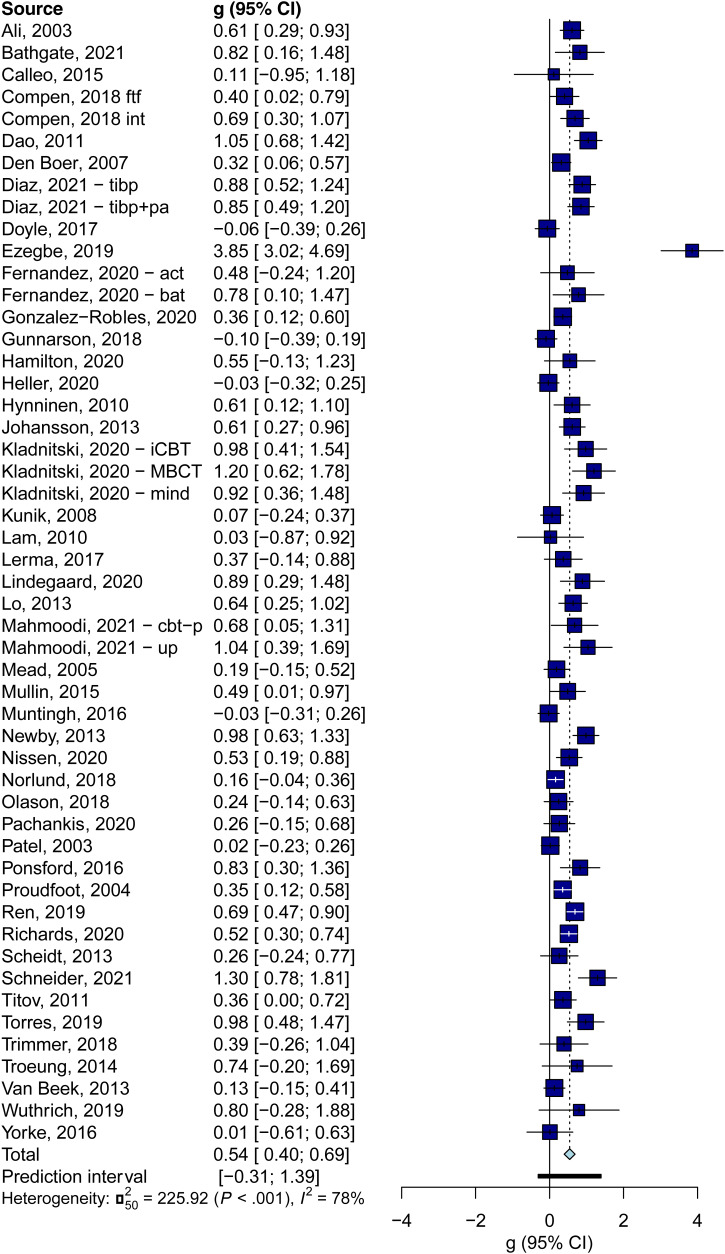
Forest plot of trials of transdiagnostic treatments for depression and anxiety.

The results of the main analyses are reported in Table 2 and the forest plot is presented in Fig. 2. When pooling all effect sizes indicating depression and anxiety in one analysis, the overall effect size for the 51 comparisons was *g* = 0.54 (95% CI 0.40–0.69), with high heterogeneity (*I*^2^ = 78; 95% CI 71–83), and a broad PI (−0.31–1.39). The NNT was 5.87. Most sensitivity analyses resulted in comparable outcomes (Table 2). When 9 outliers were removed the effect size remained comparable, but heterogeneity was low to moderate (*I*^2^ = 44; 95% CI 19–61) and the PI was narrower and did not include zero (0.18–0.96).

**Table 2.** Effects of transdiagnostic treatments of depression and anxiety

Outcomes	*k*	*g*	CI	*I*^2^	CI	Prediction interval	NNT
Depression and anxiety combined							
All comparisons (effect sizes combined)	51	0.54	0.40–0.69	78	71–83	−0.31–1.39	5.87
All studies (effect sizes combined)	45	0.51	0.35–0.67	79	72–84	−0.37–1.39	6.31
Three-Level Model (CHE)1	122	0.57	0.41–0.72	83	–	−0.41–1.54	5.60
One effect size per study (lowest)	45	0.37	0.22–0.52	71	60–78	−0.40–1.14	9.18
One effect size per study (highest)	45	0.67	0.44–0.89	79	72–84	−0.42–1.75	4.62
Outliers removed2	42	0.57	0.48–0.66	44	19–61	0.18–0.96	5.56
Influence Analysis3	50	0.49	0.38–0.59	70	60–77	−0.11–1.09	6.66
Adjusted for publication bias	66	0.33	0.15–0.51	85	81–87	−0.97–1.62	10.73
Only rob >3	26	0.53	0.38–0.69	76	66–84	−0.16–1.23	5.97
All effect sizes (assuming independence)	122	0.50	0.39–0.60	74	68–78	−0.32–1.31	6.53
Depression only							
All comparisons (effect sizes combined)	47	0.61	0.40–0.82	78	71–83	−0.39–1.61	5.11
All studies (effect sizes combined)	42	0.58	0.35–0.81	79	72–84	−0.47–1.62	5.44
Three-Level Model (CHE)1	57	0.61	0.44–0.78	83	–	−0.39–1.61	5.11
One effect size per study (lowest)	42	0.55	0.32–0.78	77	70–83	−0.50–1.61	5.72
One effect size per study (highest)	42	0.61	0.38–0.84	77	69–83	−0.44–1.65	5.15
Outliers removed4	41	0.62	0.51–0.72	46	22–63	0.16–1.08	5.04
Influence Analysis5	46	0.53	0.42–0.65	65	52–74	−0.10–1.17	6.00
Adjusted for publication bias	61	0.37	0.10–0.64	84	80–87	−1.41–2.15	9.42
Only rob >3	24	0.58	0.41–0.75	73	59–82	−0.14–1.30	5.44
All effect sizes (assuming independence)	57	0.54	0.37–0.72	76	70–82	−0.39–1.48	5.85
Anxiety only							
All comparisons (effect sizes combined)	46	0.54	0.37–0.71	75	67–81	−0.4–1.47	5.92
All studies (effect sizes combined)	41	0.50	0.32–0.69	76	68–83	−0.46–1.47	6.41
Three-Level Model (CHE)1	57	0.54	0.38–0.70	80	–	−0.39–1.47	5.90
One effect size per study (lowest)	41	0.47	0.29–0.66	76	67–82	−0.51–1.45	6.89
One effect size per study (highest)	41	0.53	0.34–0.72	76	67–82	−0.45–1.51	6.02
Outliers removed6	39	0.48	0.37–0.58	40	12–59	0.07–0.88	6.81
Influence Analysis7	45	0.47	0.35–0.60	66	53–75	−0.2–1.15	6.87
Adjusted for publication bias	57	0.33	0.12–0.54	82	78–86	−1.07–1.72	10.73
Only rob >3	24	0.49	0.30–0.68	75	62–83	−0.3–1.29	6.58
All effect sizes (assuming independence)	57	0.48	0.34–0.62	72	63–78	−0.37–1.33	6.79

1 The results of the multilevel model, using robust variance estimation, were almost identical to those of the three-level CHE model. Therefore we only report the results of the CHE model.

2 Excluded as outliers: Doyle, 2017; Ezegbe, 2019; Gunnarson, 2018; Heller, 2020; Kunik, 2008; Muntingh, 2016; Norlund, 2018; Patel, 2003; Schneider, 2021.

3 Excluded as influential cases: Ezegbe, 2019.

4 Excluded as outliers: Doyle, 2017; Ezegbe, 2019; Gunnarson, 2018; Heller, 2020; Kunik, 2008; Muntingh, 2016.

5 Excluded as influential cases: Ezegbe, 2019.

6 Excluded as outliers: Dao, 2011; Doyle, 2017; Ezegbe, 2019; Gunnarson, 2018; Muntingh, 2016; Norlund, 2018; Schneider, 2021.

7 Excluded as influential cases: Ezegbe, 2019.

There was considerable publication bias (Egger's test, *p* = 0.001). After adjustment the effect size was reduced considerably (*g* = 0.33; 95% CI 0.15–0.51), but heterogeneity remained very high (*I*^2^ = 85; 95% CI 81; 87; PI −0.97–1.62). The funnel plot is given in Appendix C.

We also calculated the pooled effect sizes separately for depression outcomes and for anxiety outcomes (Table 2). The results were very comparable to those found when depression and anxiety were taken together. The main effect for depression was *g* = 0.61 (95% CI 0.40–0.82; *I*^2^ = 78; 95% CI 71–83; PI −0.39–1.61; NNT = 5.11). After excluding outliers, the effects were not smaller than in the main analyses in which depression and anxiety were pooled, but heterogeneity was low to moderate (*I*^2^ = 46; 95% CI 22–63; PI 0.16–1.08). Adjustment for publication bias did result in a considerably smaller effect size (*g* = 0.37; 95% CI 0.10–0.64).

The main effect for anxiety outcomes was *g* = 0.54 (95% CI 0.37–0.71; *I*^2^ = 75; 95% CI 67–81; PI −0.40–1.47; NNT = 5.92). Excluding outliers resulted in a comparable effect size (*g* = 0.48; 95% CI 0.37–0.58), but again heterogeneity was much lower (*I*^2^ = 40; 95% CI 12–59; PI 0.07–0.88).

Unfortunately, there were very few studies that reported outcomes separately for those with a specific diagnosis at baseline and because the diagnoses differed considerably across studies, it was not possible to pool outcomes for each specific diagnosis.

### Subgroup analyses

We conducted a series of pre-planned subgroup analyses, comparing the effect sizes in studies on CBT *v.* other therapies, different treatment formats, target groups, recruitment strategies and type of control condition. The results are presented in Table 3. None of these subgroup analyses indicated a significant difference between subgroups, except for type of control condition. Waiting list control groups showed the largest effects (*g* = 0.79; 95% CI 0.48–1.10), usual care somewhat smaller effects (*g* = 0.44; 95% CI 0.29–0.59) and the category of ‘other’ control conditions indicated the smallest (non-significant) effect (*g* = 0.14; 95% CI −0.21 to 0.49). The difference was significant (*p* = 0.003).

**Table 3.** Subgroup analyses

		*n*_comp_	*g*	CI	*I*^2^	CI	NNT	*p*
Therapy	CBT	31	0.59	0.36–0.82	80	72–86	5.32	0.50
	Other	20	0.49	0.31–0.67	75	62–84	6.61	
Format	Individual	14	0.37	0.14–0.59	73	54–84	9.13	0.15
	Group	13	0.74	0.18–1.30	85	75–91	4.08	
	Guided self-help	19	0.60	0.42–0.77	74	59–83	5.22	
	Other	5	0.30	−0.16 to 0.76	48	0–81	11.56	
Target group	Adults	18	0.49	0.3–0.67	77	64–85	6.61	0.54
	General medical	22	0.47	0.31–0.63	67	48–79	6.94	
	Other	11	0.82	0.14–1.51	88	81–93	3.61	
Recruitment	Community	20	0.60	0.42–0.79	74	60–83	5.22	0.20
	Clinical	13	0.39	0.19–0.58	63	32–80	8.60	
	Other	18	0.60	0.19–1.00	85	77–90	5.22	
Control	Care-as-usual	25	0.44	0.29–0.59	72	58–81	7.49	0.003
	Waitlist	21	0.79	0.48–1.10	77	66–85	3.78	
	Other control	5	0.14	−0.21 to 0.49	59	0–85	26.47	
Publication year	2003–2010	8	0.28	0.09–0.46	47	0–76	12.48	0.01
	2011–2015	10	0.56	0.32–0.8	64	28–82	5.66	
	2016–2021	33	0.63	0.4–0.85	82	75–87	4.93	
OCD (<10%) incl	Yes	6	0.81	0.49–1.12	64	13–85	3.67	0.04
	No	45	0.50	0.34–0.67	78	71–83	6.46	

Because most studies were relatively new, and there appeared to be a steady increase in number of trials over the past years, we conducted a posthoc subgroup analysis to examine whether the effects differed across three categories of publication year (2003–2010; 2011–2015; 2016–2022). The results are presented in Table 3. We did indeed find a significant difference between subgroups, with the smallest effects for studies published between 2003 and 2010 (*p* = 0.01). A posthoc metaregression analysis with publication year as predictor of the effect size did not find a significant association (*p* = 0.18).

As indicated, we also conducted a posthoc subgroup analysis in which we compared studies in which some patients with OCD were included with the other studies. We did find a small, but significant difference between the effect sizes in studies in which some participants with OCD were included (*g* = 0.81; 95% CI 0.49–1.12) and studies without such participants (*g* = 0.50; 95% CI 0.34–0.67; *p* for difference = 0.04).

### Longer-term outcomes

The longer-term outcomes are reported in Appendix D (6 months post-randomisation) and Appendix E (12 months post-randomisation). At 6 months, there were 12 comparisons from 10 studies available. The main effect pooling anxiety and depression outcomes was comparable to the effect at post-test (*g* = 0.52; 95% CI 0.21–0.83; NNT = 6.13) with very high heterogeneity (*I*^2^ = 86; 95% CI 78–91; PI = −0.51–1.55). Removal of outliers did not substantially change the effect size or heterogeneity. However, after adjustment for publication bias, the effects were considerably smaller and not significant anymore (*g* = 0.28; 95% CI −0.07; 0.63; NNT = 12.48) and heterogeneity was very high (*I*^2^ = 88; 95% CI 81–92; PI = −1.04–1.61). The effects were also smaller and not significant for the subset of studies with low risk of bias (*g* = 0.20; 95% CI −0.08 to 0.49; NNT = 17.69) with moderate to high heterogeneity (*I*^2^ = 66; 95% CI 11–87; PI = −0.47–0.88). These non-significant findings may be related to lower statistical power.

The effects at 6 months for depression separately and for anxiety separately were comparable to the main outcomes at 6 months, except that the effects for depression were still significant after adjustment for publication bias.

At 12 months follow-up, data were available for 6 studies (and comparisons). The pooled effect size was zero (*g* = 0.00; 95% CI −0.24–0.24; NNT>100) with moderate heterogeneity (*I*^2^ = 41; 95% CI 0–77; PI = −0.35–0.35). All other analyses at 12 months follow-up had comparable outcomes. Too few studies provided data at longer term follow-up for further analyses.

## Discussion

In the past 10 years there has been a strong increase in numbers of randomised trials examining the effects of transdiagnostic interventions aimed at depression or anxiety. We conducted the first comprehensive meta-analysis of the trials in this emerging field. We found that they have moderate effects on depression and anxiety (effect sizes ranging from *g* = 0.52 to 0.63). Heterogeneity was high in all analyses, but the effects were robust across an extensive series of sensitivity analyses and were still significant after adjustment for publication bias and when limiting to studies with low risk of bias. Removal of outliers resulted in lower levels of heterogeneity. These findings are in line with previous, more narrow meta-analyses of transdiagnostic treatments of depression and anxiety, which overall also found moderate to large effects of such treatments (Andersen et al., 2016; Newby et al., 2015, 2016; Păsărelu et al., 2017; Pearl & Norton, 2017; Reinholt & Krogh, [Bibr ref40][Bibr ref40]; Sakiris & Berle, 2019; Thompson et al., 2021).

These findings suggest that these transdiagnostic treatments are effective in the treatment of depression and anxiety. These effects remain significant at six months after baseline. However, these longer term effects are uncertain because adjustment for publication bias and analyses limited to studies with low risk of bias were not significant anymore in most analyses. At one year after baseline we found no indication anymore that the interventions significantly reduced depression and anxiety.

Almost 80% of the 45 trials in this meta-analysis were published in the last 10 years and only 8 trials were published before 2010. This indicates that focusing on these transdiagnostic treatments is clearly a trend and it can be expected that in the coming years many more of such trials will be published. Considering the high comorbidity between depression and anxiety, and the common elements in treatments of both disorders, trials examining such transdiagnostic treatments makes complete sense. A considerable number of trials (20) were aimed at people with comorbid general medical disorders. In such settings, transdiagnostic treatments are much more practical than developing treatments separately for depression and anxiety.

Our subgroup analyses suggested no significant differences between CBT and other therapies, treatment formats, target groups and recruitment strategies. We did find significant differences between studies using different types of control conditions, studies published across different periods and studies in which OCD was included *v.* studies in which this was not the case. Heterogeneity remained moderate to high in all examined subgroups. Subgroup analyses always have to be considered with caution because they are typically highly underpowered (Cuijpers et al., 2021) and other unmeasured variables may influence the outcomes. It seems unlikely that the difference between studies with and without OCD patients can indeed be attributed to the proportion of OCD patients, because this proportion was less than 10% in all studies. The possibility of biased results is however, not impossible. The difference between types of control groups is well-established in other meta-analyses (e.g. Cuijpers et al., 2021) and may be related to inflated effect sizes in waitlist controlled trials. The association with publication year may indicate an improvement of treatments over time, although this may very well be a chance finding.

This meta-analysis illustrates one of the most important advantages of ‘Meta-analytic Research Domains’ (MARDs; Cuijpers *et al*. 2022a, 2022b). A MARD is a living systematic review that does not focus on one specific research question, but searches for trials in a full research domain, in our case psychotherapy for depression. Because we search for all trials on psychotherapy for depression, regardless of the comparator, we also see these trials focusing on transdiagnostic treatment. A ‘normal’ search does not identify these trials, because most trials are not explicitly identified as transdiagnostic treatment. It requires searches covering this whole domain to find these trials, and to notify this upcoming trend in the field.

This meta-analysis has several limitations that must be taken into account when interpreting the results. First, the quality of many included studies was not optimal. Furthermore, most studies did not provide longer term follow-up outcomes, while this is an essential outcome. Heterogeneity was also very high in many analyses, making it uncertain what the actual outcomes of these interventions are. Only few studies reported outcomes for those with specific diagnoses or problems at baseline. This means that these outcomes are still unclear.

Despite these limitations, however, we can conclude that transdiagnostic treatments of depression and/or anxiety are probably effective at the short term. More high-quality research is needed to verify these findings and to examine the effects at the longer term.

## Supplementary material

For supplementary material accompanying this paper visit https://doi.org/10.1017/S0033291722003841. click here to view supplementary material
