# Supplementary material for: Transdiagnostic treatment of depression and anxiety: a meta-analysis
Source: Psychol Med. 2023 Jan 6;53(14):6535–46. doi: 10.1017/S0033291722003841 (PMC10600931; doi:10.1017/S0033291722003841)
Supplement: Supplementary file 1 [file S0033291722003841sup001.docx]

Appendix A. Search strings for four bibliographical databases

PubMed

Psychotherapy [MH] OR psychotherap*[All Fields] OR cbt[All Fields] OR "behavior therapies"[All Fields] OR "behavior therapy"[All Fields] OR "behavior therapeutic"[All Fields] OR "behavior therapeutical"[All Fields] OR "behavior therapeutics"[All Fields] OR "behavior therapeutist"[all Fields] OR "behavior therapeutists"[All Fields] OR "behavior treatment"[All Fields] OR "behavior treatments"[All Fields] OR "behaviors therapies"[All Fields] OR "behaviors therapy"[All Fields] OR "behaviors therapeutics"[All Fields] OR "behaviors therapeutic"[All Fields] OR "behaviors therapeutical"[All Fields] OR "behaviors therapeutist"[All Fields] OR "behaviors therapeutists"[All Fields] OR "behaviors treatment"[All Fields] OR "behaviors treatments"[All Fields] OR "behavioral therapies"[All Fields] OR "behavioral therapy"[All Fields] OR "behavioral therapeutics"[All Fields] OR "behavioral therapeutic"[All Fields] OR "behavioral therapeutical"[All Fields] OR "behavioral therapeutist"[All Fields] OR "behavioral therapeutists"[All Fields] OR "behavioral treatment"[All Fields] OR "behavioral treatments"[All Fields] OR "behaviour therapies"[All Fields] OR "behaviour therapy"[All Fields] OR "behaviour therapeutic"[All Fields] OR "behaviour therapeutical"[All Fields] OR "behaviour therapeutics"[All Fields] OR "behaviour therapeutist"[all Fields] OR "behaviour therapeutists"[All Fields] OR "behaviour treatment"[All Fields] OR "behaviour treatments"[All Fields] OR "behaviours therapies"[All Fields] OR "behaviours therapy"[All Fields] OR "behaviours therapeutics"[All Fields] OR "behaviours therapeutic"[All Fields] OR "behaviours therapeutical"[All Fields] OR "behaviours therapeutist"[All Fields] OR "behaviours therapeutists"[All Fields] OR "behaviours treatment"[All Fields] OR "behaviours treatments"[All Fields] OR "behavioural therapies"[All Fields] OR "behavioural therapy"[All Fields] OR "behavioural therapeutics"[All Fields] OR "behavioural therapeutic"[All Fields] OR "behavioural therapeutical"[All Fields] OR "behavioural therapeutist"[All Fields] OR "behavioural therapeutists"[All Fields] OR "behavioural treatment"[All Fields] OR "behavioural treatments"[All Fields] OR "cognition therapies"[All Fields] OR "cognition therapie"[All Fields] OR "cognition therapy"[All Fields] OR "cognition therapeutical"[All Fields] OR "cognition therapeutic"[All Fields] OR "cognition therapeutics"[All Fields] OR "cognition therapeutist"[All Fields] OR "cognition therapeutists"[All Fields] OR "cognition treatment"[All Fields] OR "cognition treatments"[All Fields] OR psychodynamic[All Fields] OR Psychoanalysis[MH] OR psychoanalysis[All Fields] OR psychoanalytic*[All Fields] OR counselling[All Fields] OR counseling[All Fields] OR Counseling[MH] OR "problem-solving"[All Fields] OR mindfulness[All Fields] OR (acceptance[All Fields] AND commitment[All Fields] ) OR "assertiveness training"[All Fields] OR "behavior activation"[All Fields] OR "behaviors activation"[All Fields] OR "behavioral activation"[All Fields] OR "cognitive therapies"[All Fields] OR "cognitive therapy"[All Fields] OR "cognitive therapeutic"[All Fields] OR "cognitive therapeutics"[All Fields] OR "cognitive therapeutical"[All Fields] OR "cognitive therapeutist"[All Fields] OR "cognitive therapeutists"[All Fields] OR "cognitive treatment"[All Fields] OR "cognitive treatments"[All Fields] OR "cognitive restructuring"[All Fields] OR (("compassion-focused"[All Fields] OR "compassion-focussed"[All Fields]) AND (therapy[SH] OR therapies[All Fields] OR therapy[All Fields] OR therape*[All Fields] OR therapis*[All Fields]OR Therapeutics [OR treatment*[All Fields])) OR ((therapy[SH] OR therapies[All Fields] OR therapy [All Fields] OR therape*[All Fields] OR therapis*[All Fields] OR Therapeutics[MH] OR treatment*[All Fields]) AND constructivist*[All Fields]) OR "metacognitive therapies"[All Fields] OR "metacognitive therapy"[All Fields] OR "metacognitive therapeutic"[All Fields] OR "metacognitive therapeutics"[All Fields] OR "metacognitive therapeutical"[All Fields] OR "metacognitive therapeutist"[All Fields] OR "metacognitive therapeutists"[All Fields] OR "metacognitive treatment"[All Fields] OR "metacognitive treatments"[All Fields] OR "meta-cognitive therapies"[All Fields] OR "meta-cognitive therapy"[All Fields] OR "meta-cognitive therapeutic"[All Fields] OR "meta-cognitive therapeutics"[All Fields] OR "meta-cognitive therapeutical"[All Fields] OR "meta-cognitive therapeutist"[All Fields] OR "meta-cognitive therapeutists"[All Fields] OR "meta-cognitive treatment"[All Fields] OR "meta-cognitive treatments"[All Fields] OR "solution-focused therapies"[All Fields] OR "solution-focused therapy"[All Fields] OR "solution-focused therapeutic"[All Fields] OR "solution-focused therapeutics"[All Fields] OR "solution-focused therapeutical"[All Fields] OR "solution focused therapies"[All Fields] OR "solution focused therapy"[All Fields] OR "solution focused therapeutic"[All Fields] OR "solution focused therapeutics"[All Fields] OR "solution focused therapeutical"[All Fields]OR "solution-focussed therapies"[All Fields] OR "solution-focussed therapy"[All Fields] OR "solution-focussed therapeutic"[All Fields] OR "solution-focussed therapeutics"[All Fields] OR "solution-focussed therapeutical"[All Fields]OR "solution focussed therapies"[All Fields] OR "solution focussed therapy"[All Fields] OR "solution focussed therapeutic"[All Fields] OR "solution focussed therapeutics"[All Fields] OR "solution focussed therapeutical"[All Fields] OR "self-control therapies"[All Fields] OR "self-control therapy"[All Fields] OR "self-control therapeutics"[All Fields] OR "self-control therapeutical"[All Fields] OR "self-control therapeutic"[All Fields] OR "self- control training"[All Fields] OR "self-control trainings"[All Fields] OR "self control therapies"[All Fields] OR "self control therapy"[All Fields] OR "self control therapeutics"[All Fields] OR "self control therapeutical"[All Fields] OR "self control therapeutic"[All Fields] OR "self control training"[All Fields] OR "self control trainings"[All Fields]

AND

(Depressive Disorder[MH] OR Depression[MH]OR dysthymi*[All Fields] OR "affective disorder"[All Fields]OR "affective disorders"[All Fields] OR "mood disorder"[All Fields] OR "mood disorders"[All Fields] OR depression*[All Fields] OR depressive*[All Fields] OR "dysthymic disorder"[MeSH Terms])

Limits: RCTs

Embase

#1

'psychotherapy'/exp OR 'psychotherapy' OR 'psychotherapies' OR 'psychotherapeutics' OR 'psychotherapeutical' OR 'cognitive therapy'/exp OR 'cognitive behavior therapy'/exp OR 'behavior therapy'/exp OR ́cbt ́ OR ́cognitive behavioural therapy ́ OR ́cognitive behavioural therapies ́ OR cognitive behavioral therapy ́ OR 'cognitive behavioral therapies' OR 'behavior therapy' OR 'behavior therapies' OR 'behaviour therapy' OR 'behaviour therapies' OR 'cognition therapy' OR 'cognitive therapies' OR 'cognitive therapy' OR 'cognitive therapeutic' OR 'cognitive therapeutics' OR 'cognitive therapeutical' OR 'cognitive therapeutist' OR 'cognitive therapeutists' OR 'cognitive treatment' OR 'cognitive treatments' OR 'cognitive restructuring' OR 'cognition therapies' OR 'cognition therapie' OR 'cognition therapeutical' OR 'cognition therapeutic' OR 'cognition therapeutics' OR 'cognition therapeutist' OR 'cognition therapeutists' OR 'cognition treatment' OR 'cognition treatments' OR 'behavior therapeutic' OR 'behavior therapeutical' OR 'behavior therapeutics' OR 'behavior therapeutist' OR 'behavior therapeutists' OR 'behavior treatment' OR 'behavior treatments' OR 'behaviors therapies' OR 'behaviors therapy' OR 'behaviors therapeutics' OR 'behaviors therapeutic' OR 'behaviors therapeutical' OR 'behaviors therapeutist' OR 'behaviors therapeutists' OR 'behaviors treatment' OR 'behaviors treatments' OR 'behavioral therapies' OR 'behavioral therapy' OR 'behavioral therapeutics' OR 'behavioral therapeutic' OR 'behavioral therapeutical' OR 'behavioral therapeutist' OR 'behavioral therapeutists' OR 'behavioral treatment' OR 'behavioral treatments' OR 'behaviour therapeutic' OR 'behaviour therapeutical' OR 'behaviour therapeutics' OR 'behaviour therapeutist' OR 'behaviour therapeutists' OR 'behaviour treatment' OR 'behaviour treatments' OR 'behaviours therapies' OR 'behaviours therapy' OR 'behaviours therapeutics' OR 'behaviours therapeutic' OR 'behaviours therapeutical' OR 'behaviours therapeutist' OR 'behaviours therapeutists' OR 'behaviours treatment' OR 'behaviours treatments' OR 'behavioural therapies' OR 'behavioural therapy' OR 'behavioural therapeutics' OR 'behavioural therapeutic' OR 'behavioural therapeutical' OR 'behavioural therapeutist' OR 'behavioural therapeutists' OR 'behavioural treatment' OR 'behavioural treatments' OR 'behavior activation' OR 'behaviors activation' OR 'behavioral activation' OR 'behaviour activation' OR 'behaviours activation' OR 'behavioural activation' OR 'psychoanalytic therapy'/exp OR 'psychodynamic' OR 'psychodynamical' OR 'psychoanalysis' OR 'psychoanalytical' OR 'counselling'/exp OR 'counseling'/exp OR 'counselling' OR 'counseling' OR 'problem-solving' OR 'problem solving' OR 'supportive therapy' OR 'metacognitive therapy' OR 'metacognitive therapies' OR 'metacognitive therapeutic' OR 'metacognitive therapeutics' OR 'metacognitive therapeutical' OR 'metacognitive therapeutist' OR 'metacognitive therapeutists' OR 'metacognitive treatment' OR 'metacognitive treatments' OR 'meta-cognitive therapy' OR 'meta-cognitive therapies' OR 'meta-cognitive therapeutic' OR 'meta-cognitive therapeutics' OR 'meta-cognitive therapeutical' OR 'meta-cognitive therapeutist' OR 'meta-cognitive therapeutists' OR 'meta-cognitive treatment' OR 'meta-cognitive treatments' OR 'solution-focused therapies' OR 'solution focused therapies' OR 'solution-focussed therapies' OR 'solution focused therapies' OR 'solution- focused therapy' OR 'solution focused therapy' OR 'solution-focussed therapy' OR 'solution focused therapy' OR 'solution-focused therapeutic' OR 'solution focused therapeutic' OR 'solution-focussed therapeutic' OR 'solution focussed therapeutic' OR 'solution-focused therapeutics' OR 'solution focused therapeutics' OR 'solution-focussed therapeutics' OR 'solution focused therapeutics' OR 'solution-focused therapeutical' OR 'solution focused therapeutical' OR 'solution-focussed therapeutical' OR 'solution focused therapeutical' OR 'self-control therapies' OR 'self control therapies' OR 'self-control therapy' OR 'self control therapy' OR 'self-control therapeutics' OR 'self control therapeutics' OR 'self-control therapeutical' OR 'self control therapeutical' OR 'self-control therapeutic' OR 'self control therapeutic' OR 'self-control training' OR 'self control training' OR 'self control trainings' OR 'self-control trainings' OR 'mindfulness' OR 'acceptance commitment' OR 'acceptance and commitment' OR 'assertiveness training'

#2

'compassion-focused' OR 'compassion-focussed' OR 'compassion focused' OR 'compassion focussed' OR 'constructivist' OR 'constructivists'

#3

'therapies' OR 'therapy' OR 'therapeutics' OR 'therapist' OR 'treatment' OR 'treatments'

#4

Combine: #2 AND #3

#5: #1 OR #4

#6

'depressive disorder'/exp OR 'depression'/exp OR 'depressive' OR 'major depression'/exp OR 'major depressive disorder'/exp OR 'depression' OR 'depressions' OR 'depressive' OR 'dysthymic disorder'/exp OR 'dysthymic disorder' OR 'dysthymia'/exp OR 'dysthymic' OR 'mood disorder'/exp OR 'affective disorder'/exp OR 'affective disorder' OR 'affective disorders' OR 'mood disorder' OR 'mood disorders'

Combine: #5 AND #6

Limits: RCTs

PsycINFO

(DE "Psychotherapy" OR "Psychotherapy" OR "psychotherapies" OR "psychotherapeutic" OR "psychotherapeutical" OR "psychotherapeutics" OR DE "Behavior Therapy" OR DE "Cognitive Behavior Therapy" OR "CBT" OR "behavior therapies" OR "behavior therapy" OR "behavior therapeutic" OR "behavior therapeutical" OR "behavior therapeutics" OR "behavior therapeutist" OR "behavior therapeutists" OR "behavior treatment" OR "behavior treatments" OR "behaviors therapies" OR "behaviors therapy" OR "behaviors therapeutics" OR "behaviors therapeutic" OR "behaviors therapeutical" OR "behaviors therapeutist" OR "behaviors therapeutists" OR "behaviors treatment" OR "behaviors treatments" OR "behavioral therapies" OR "behavioral therapy" OR "behavioral therapeutics" OR "behavioral therapeutic" OR "behavioral therapeutical" OR "behavioral therapeutist" OR "behavioral therapeutists" OR "behavioral treatment" OR "behavioral treatments" OR "behaviour therapies" OR "behaviour therapy" OR "behaviour therapeutic" OR "behaviour therapeutical" OR "behaviour therapeutics" OR "behaviour therapeutist" OR "behaviour therapeutists" OR "behaviour treatment" OR "behaviour treatments" OR "behaviours therapies" OR "behaviours therapy" OR "behaviours therapeutics" OR "behaviours therapeutic" OR "behaviours therapeutical" OR "behaviours therapeutist" OR "behaviours therapeutists" OR "behaviours treatment" OR "behaviours treatments" OR "behavioural therapies" OR "behavioural therapy" OR "behavioural therapeutics" OR "behavioural therapeutic" OR "behavioural therapeutical" OR "behavioural therapeutist" OR "behavioural therapeutists" OR "behavioural treatment" OR "behavioural treatments" OR "cognition therapies" OR "cognition therapie" OR "cognition therapy" OR "cognition therapeutical" OR "cognition therapeutic" OR "cognition therapeutics" OR "cognition therapeutist" OR "cognition therapeutists" OR "cognition treatment" OR "cognition treatments" OR "cognitive therapies" OR "cognitive therapy" OR "cognitive therapeutic" OR "cognitive therapeutics" OR "cognitive therapeutical" OR "cognitive therapeutist" OR "cognitive therapeutists" OR "cognitive treatment" OR "cognitive treatments" OR "cognitive restructuring" OR DE "Emotion Focused Therapy" OR DE "Psychoanalysis" OR "psychoanalysis" OR "psychoanalytic" OR "psychoanalytical "OR DE "Psychodynamic Psychotherapy" OR "psychodynamic" OR DE "Psychotherapeutic Counseling" OR "counselling" OR "counseling" OR "problem-solving" OR "problem solving" OR "mindfulness" OR ("acceptance" AND "commitment") OR "assertiveness training" OR "behavior activation" OR "behaviors activation" OR "behavioral activation" OR "behaviour activation" OR "behaviours activation" OR "behavioural activation" OR "metacognitive therapies" OR "metacognitive therapy" OR "metacognitive therapeutic" OR "metacognitive therapeutics" OR "metacognitive therapeutical" OR "metacognitive therapeutist" OR "metacognitive therapeutists" OR "metacognitive treatment" OR "metacognitive treatments" OR "meta-cognitive therapies" OR "meta-cognitive therapy" OR "meta-cognitive therapeutic" OR "meta-cognitive therapeutics" OR "meta-cognitive therapeutical" OR "meta-cognitive therapeutist" OR "meta-cognitive therapeutists" OR "meta-cognitive treatment" OR "meta-cognitive treatments" OR DE "Solution Focused Therapy" OR "solution- focused therapies" OR "solution-focused therapy" OR "solution-focused therapeutic" OR "solution-focused therapeutics" OR "solution-focused therapeutical" OR "solution-focussed therapies" OR "solution-focussed therapy" OR "solution-focussed therapeutic" OR "solution-focussed therapeutics" OR "solution-focussed therapeutical" OR "solution focused therapies" OR "solution focused therapy" OR "solution focused therapeutic" OR "solution focused therapeutics" OR "solution focused therapeutical" OR "solution focussed therapies" OR "solution focussed therapy" OR "solution focussed therapeutic" OR "solution focussed therapeutics" OR "solution focussed therapeutical" OR "self- control therapies" OR "self-control therapy" OR "self-control therapeutics" OR "self-control therapeutical" OR "self- control therapeutic" OR "self-control training" OR "self-control trainings" OR "self control therapies" OR "self control therapy" OR "self control therapeutics" OR "self control therapeutical" OR "self control therapeutic" OR "self control training" OR "self control trainings" OR (("compassion-focused" OR "compassion-focussed" OR "compassion focused" OR "compassion focussed") AND ("therapies" OR "therapy" OR "therapie" OR "therapist" OR "therapists" OR "therapeut" OR "treatment" OR "treatments")) OR ("constructivist" AND ("therapies" OR "therapy" OR "therapie" OR "therapist" OR "therapists" OR "therapeut" OR "treatment" OR "treatments")))

AND

(DE "Depression (Emotion)" "depressive disorder" OR "depression" OR "depressions" OR "depressive" OR DE "Major Depression" OR "major depression" OR "major depressive disorder" OR DE "Dysthymic Disorder" OR "Dysthymia" OR " dysthymic disorder" OR DE "Affective Disorders" OR "Affective Disorder" OR "affective disorders" OR "Mood Disorder" OR "Mood disorders")

Limits: Methodology is ME=(treatment outcome/clinical trial): papers (December 2014)

Cochrane

#1 MeSH descriptor: [Depressive Disorder] explode all trees : 6, 777

#2 ''depress*'' (Word variations have been searched) : 51, 768

#3 #1or#2 :51,783

#4 ''major depressive disorder'' (Word variations have been searched) : 5, 435

#5 #3or#4 :51,783

#6 MeSH descriptor: [Dysthymic Disorder] explode all trees : 129

#7 ''dysthymi*'' (Word variations have been searched) : 649

#8 #6or#7 :649

#9 #5or#8 :51,800

#10 ''mood disorder'' (Word variations have been searched) :4, 034

#11 ''affective disorder'' (Word variations have been searched) : 2, 882

#12 #10or#11:6,055

#13 #9or#12:53,227

#14 MeSH descriptor: [Psychotherapy] explode all trees : 13, 568

#15 ''psychotherap*'' (Word variations have been searched) : 7, 758

#16 ''CBT'' (Word variations have been searched) : 2, 029

#17 ''Cognitive Behav* therap* (Word variations have been searched) : 8, 893

#18 #14or#15or#16or#17 :20,795

#19 'psychodynamic'' (Word variations have been searched) : 469

#20 MeSH descriptor: [Psychoanalysis] explode all trees : 13

#21 ''psychoanaly*'' (Word variations have been searched) : 345

#22 MeSH descriptor: [Counseling] explode all trees : 2, 783

#23 ''counseling*'' (Word variations have been searched) : 6, 913

#24 ''problem solving'' (Word variations have been searched) : 2, 867

#25 #18or#19or#20or#21or#22or#23or#24 :28,149

#26 ''acceptance commitment'' (Word variations have been searched) : 168

#27 ''assertiveness training'' (Word variations have been searched) :231

#28 ''behavior activation'' (Word variations have been searched) : 663

#29 ''mindfulness'' (Word variations have been searched) : 466

#30 ''metacognitive therap*'' (Word variations have been searched) :56

#31 ''solution focused therap*'' (Word variations have been searched) :858

#32 ''self control training'' (Word variations have been searched): 5850

#33 #25or#26or#27or#28or#29or#30or#31or#32 :32,748

#34 ''Randomized Controlled Trial'':ti,ab,kw (Word variations have been searched) : 120, 901

#35 #13 and #33 and #34 in Trials: 4,614

Limit: publication year: 1970-2018

Appendix B. References of included studies

Ali BS, Rahbar MH, Naeem S, Gul A, Mubeen S, Iqbal A. The effectiveness of counseling on anxiety and depression by minimally trained counselors: a randomized controlled trial. American journal of psychotherapy. 2003;57(3):324-36.

Bathgate CJ, Kilbourn KM, Murphy NH, Wamboldt FS, Holm KE. Pilot RCT of a telehealth intervention to reduce symptoms of depression and anxiety in adults with cystic fibrosis. Journal of Cystic Fibrosis. 2021.

Calleo JS, Amspoker AB, Sarwar AI, Kunik ME, Jankovic J, Marsh L, et al. A Pilot Study of a Cognitive-Behavioral Treatment for Anxiety and Depression in Patients with Parkinson Disease. Journal of Geriatric Psychiatry and Neurology. 2015;28(3):210-7.

Compen F, Bisseling E, Schellekens M, Donders R, Carlson L, van der Lee M, et al. Face-to-face and internet-based mindfulness-based cognitive therapy compared with treatment as usual in reducing psychological distress in patients with cancer: A multicenter randomized controlled trial. Journal of Clinical Oncology. 2018;36(23):2413-21.

Dao TK, Youssef NA, Armsworth M, Wear E, Papathopoulos KN, Gopaldas R. Randomized controlled trial of brief cognitive behavioral intervention for depression and anxiety symptoms preoperatively in patients undergoing coronary artery bypass graft surgery. The Journal of thoracic and cardiovascular surgery. 2011;142(3):e109-15.

den Boer PC, Wiersma D, Ten Vaarwerk I, Span MM, Stant AD, Van den Bosch RJ. Cognitive self-therapy for chronic depression and anxiety: a multi-centre randomized controlled study. Psychological medicine. 2007;37(3):329-39.

Díaz-García A, González-Robles A, García-Palacios A, Fernández-Álvarez J, Castilla D, Bretón JM, et al. Negative and Positive Affect Regulation in a Transdiagnostic Internet-Based Protocol for Emotional Disorders: Randomized Controlled Trial. J Med Internet Res. 2021;23(2):e21335.

Doyle C, Bhar S, Fearn M, Ames D, Osborne D, You E, et al. The impact of telephone-delivered cognitive behaviour therapy and befriending on mood disorders in people with chronic obstructive pulmonary disease: a randomized controlled trial. British journal of health psychology [Internet]. 2017. Available from: http://onlinelibrary.wiley.com/o/cochrane/clcentral/articles/597/CN-01402597/frame.html

Ezegbe BN, Eseadi C, Ede MO, Igbo JN, Anyanwu JI, Ede KR, et al. Impacts of cognitive-behavioral intervention on anxiety and depression among social science education students: A randomized controlled trial. Medicine (Baltimore). 2019;98(15):e14935-e.

Fernández-Rodríguez C, González-Fernández S, Coto-Lesmes R, Pedrosa I. Behavioral Activation and Acceptance and Commitment Therapy in the Treatment of Anxiety and Depression in Cancer Survivors: A Randomized Clinical Trial. Behavior modification. 2020:145445520916441.

Gunnarsson BA, Wagman P, Hedin K, Hakansson C. Treatment of depression and/or anxiety - outcomes of a randomised controlled trial of the tree theme method(R) versus regular occupational therapy. BMC Psychol. 2018;6(1):25.

González-Robles, A., Díaz-García, A., García-Palacios, A., Roca, P., Ramos-Quiroga, J.A., & Botella, C. (2020). Effectiveness of a Transdiagnostic Guided Internet-Delivered Protocol for Emotional Disorders Versus Treatment as Usual in Specialized Care: Randomized Controlled Trial. Journal of Medical Internet Research, 22, e18220

Hamilton J, Saxon D, Best E, Glover V, Walters SJ, Kerr IB. A randomised, controlled pilot study of cognitive analytic therapy (CAT) for stressed pregnant women with underlying anxiety and depression in a routine health service setting. Clinical psychology & psychotherapy. 2020.

Heller HM, Hoogendoorn AW, Honig A, Broekman BFP, van Straten A. The Effectiveness of a Guided Internet-Based Tool for the Treatment of Depression and Anxiety in Pregnancy (MamaKits Online): Randomized Controlled Trial. Journal of medical Internet research. 2020;22(3):e15172.

Hynninen MJ, Bjerke N, Pallesen S, Bakke PS, Nordhus IH. A randomized controlled trial of cognitive behavioral therapy for anxiety and depression in COPD. Respiratory medicine. 2010;104(7):986-94.

Johansson R, Bjorklund M, Hornborg C, Karlsson S, Hesser H, Ljotsson BL, et al. Affect-focused psychodynamic psychotherapy for depression and anxiety through the internet: A randomized controlled trial. PeerJ. 2013;2013(1).

Kladnitski N, Smith J, Uppal S, James MA, Allen AR, Andrews G, et al. Transdiagnostic internet-delivered CBT and mindfulness-based treatment for depression and anxiety: A randomised controlled trial. Internet Interventions. 2020;20.

Kunik ME, Veazey C, Cully JA, Souchek J, Graham DP, Hopko D, et al. COPD education and cognitive behavioral therapy group treatment for clinically significant symptoms of depression and anxiety in COPD patients: a randomized controlled trial. Psychological medicine. 2008;38(3):385-96.

Lam CL, Fong DY, Chin WY, Lee PW, Lam ET, Lo YY. Brief problem-solving treatment in primary care (PST-PC) was not more effective than placebo for elderly patients screened positive of psychological problems. International journal of geriatric psychiatry. 2010;25(10):968-80.

Lerma A, Perez-Grovas H, Bermudez L, Peralta-Pedrero ML, Robles-García R, Lerma C. Brief cognitive behavioural intervention for depression and anxiety symptoms improves quality of life in chronic haemodialysis patients. Psychology and psychotherapy. 2017;90(1):105-23.

Lindegaard T, Seaton F, Halaj A, Berg M, Kashoush F, Barchini R, et al. Internet-based cognitive behavioural therapy for depression and anxiety among Arabic-speaking individuals in Sweden: a pilot randomized controlled trial. Cognitive behaviour therapy. 2020:1-20.

Lo HH, Ng SM, Chan CL, Lam KF, Lau BH. The Chinese medicine construct "stagnation" in mind-body connection mediates the effects of mindfulness training on depression and anxiety. Complementary therapies in medicine. 2013;21(4):348-57.

Mahmoodi M, Bakhtiyari M, Masjedi Arani A, Mohammadi A, Saberi Isfeedvajani M. The comparison between CBT focused on perfectionism and CBT focused on emotion regulation for individuals with depression and anxiety disorders and dysfunctional perfectionism: A randomized controlled trial. Behavioural and Cognitive Psychotherapy. 2021;49(4):454-71.

Mead N, MacDonald W, Bower P, Lovell K, Richards D, Roberts C, et al. The clinical effectiveness of guided self-help versus waiting-list control in the management of anxiety and depression: A randomized controlled trial. Psychological Medicine. 2005;35(11):1633-43.

Mullin A, Dear BF, Karin E, Wootton BM, Staples LG, Johnston L, et al. The UniWellbeing course: A randomised controlled trial of a transdiagnostic internet-delivered cognitive behavioural therapy (CBT) programme for university students with symptoms of anxiety and depression. Internet Interventions [Internet]. 2015; 2(2 // (NHMRC) *National Health and Medical Research Council*):[128-36 pp.]. Available from: http://onlinelibrary.wiley.com/o/cochrane/clcentral/articles/428/CN-01069428/frame.html

Muntingh A. Quality of life and symptoms in patients with chronic depression and anxiety after a self-management training: A randomised controlled trial. [Dutch]. Tijdschrift voor Psychiatrie 58 (7) (pp 504-512), 2016 Date of Publication: July 2016 [Internet]. 2016. Available from: http://onlinelibrary.wiley.com/o/cochrane/clcentral/articles/365/CN-01195365/frame.html.

Newby JM, Mackenzie A, Williams AD, McIntyre K, Watts S, Wong N. Internet cognitive behavioural therapy for mixed anxiety and depression: a randomized controlled trial and evidence of effectiveness in primary care [Wellbeing 6]. Psychological Medicine [Internet]. 2013; 43(12):[2635-48 pp.]. Available from: http://onlinelibrary.wiley.com/o/cochrane/clcentral/articles/626/CN-01038626/frame.html

Nissen ER, O'Connor M, Kaldo V, Højris I, Borre M, Zachariae R, et al. Internet-delivered mindfulness-based cognitive therapy for anxiety and depression in cancer survivors: A randomized controlled trial. Psycho-oncology. 2020;29(1):68-75.

Norlund F, Wallin E, Olsson MGE, Wallert J, Burell G, Von Essen L, et al. Internet-based cognitive behavior therapy for symptoms of depression and anxiety among patients with a recent myocardial infarction: the U-CARE Heart randomized trial. European journal of preventive cardiology. 2018;25(2):S63‐S4.

Olason M, Andrason RH, Jonsdottir IH, Kristbergsdottir H, Jensen MP. Cognitive Behavioral Therapy for Depression and Anxiety in an Interdisciplinary Rehabilitation Program for Chronic Pain: a Randomized Controlled Trial with a 3-Year Follow-up. Int J Behav Med. 2018;25(1):55-66.

Pachankis JE, McConocha EM, Clark KA, Wang K, Behari K, Fetzner BK, et al. A transdiagnostic minority stress intervention for gender diverse sexual minority women's depression, anxiety, and unhealthy alcohol use: A randomized controlled trial. Journal of consulting and clinical psychology. 2020;88(7):613-30.

Patel V, Chisholm D, Rabe-Hesketh S, Dias-Saxena F, Andrew G, Mann A. Efficacy and cost-effectiveness of drug and psychological treatments for common mental disorders in general health care in Goa, India: a randomised, controlled trial. Lancet [Internet]. 2003; 361(9351):[33-9 pp.]. Available from: http://onlinelibrary.wiley.com/o/cochrane/clcentral/articles/624/CN-00412624/frame.html

Ponsford J, Lee NK, Wong D, McKay A, Haines K, Alway Y, et al. Efficacy of motivational interviewing and cognitive behavioral therapy for anxiety and depression symptoms following traumatic brain injury. Psychological medicine. 2016;46(5):1079-90.

Proudfoot J, Ryden C, Everitt B, Shapiro DA, Goldberg D, Mann A, et al. Clinical efficacy of computerised cognitive-behavioural therapy for anxiety and depression in primary care: randomised controlled trial. The British journal of psychiatry : the journal of mental science. 2004;185:46-54.

Ren W, Qiu H, Yang Y, Zhu X, Zhu C, Mao G, et al. Randomized controlled trial of cognitive behavioural therapy for depressive and anxiety symptoms in Chinese women with breast cancer. Psychiatry Research. 2019;271:52-9.

Richards D, Enrique A, Eilert N, Franklin M, Palacios J, Duffy D, et al. A pragmatic randomized waitlist-controlled effectiveness and cost-effectiveness trial of digital interventions for depression and anxiety. npj Digital Medicine. 2020;3(1).

Scheidt CE, Waller E, Endorf K, Schmidt S, Kˆnig R, Zeeck A, et al. Is brief psychodynamic psychotherapy in primary fibromyalgia syndrome with concurrent depression an effective treatment? A randomized controlled trial. General hospital psychiatry [Internet]. 2013; 35(2):[160-7 pp.]. Available from: http://onlinelibrary.wiley.com/o/cochrane/clcentral/articles/075/CN-00878075/frame.html

Schneider LH. Efficacy of Internet-delivered cognitive behavioural therapy following an acute coronary event: A randomized controlled trial: ProQuest Information & Learning; 2021.

Titov, N., Dear, B. F., Schwencke, G., Andrews, G., Johnston, L., Craske, M. G., et al. (2011). Transdiagnostic internet treatment for anxiety and depression: a randomised controlled trial. Behaviour Research and Therapy, 49, 441–452.

Torres-Platas SG, Escobar S, Belliveau C, Wu J, Sasi N, Fotso J, et al. Mindfulness-Based Cognitive Therapy Intervention for the Treatment of Late-Life Depression and Anxiety Symptoms in Primary Care: A Randomized Controlled Trial. and Psychosomatics. 2019;88(4):254-6.

Trimmer C, Tyo R, Pikard J, McKenna C, Naeem F. Low-Intensity Cognitive Behavioural Therapy-Based Music Group (CBT-Music) for the Treatment of Symptoms of Anxiety and Depression: A Feasibility Study. Behavioural and cognitive psychotherapy. 2018;46(2):168-81.

Troeung L, Egan SJ, Gasson N. A waitlist-controlled trial of group cognitive behavioural therapy for depression and anxiety in Parkinson's disease. BMC psychiatry. 2014;14(1).

van Beek MHCT, Voshaar RCO, Beek AM, van Zijderveld GA, Visser S, Speckens AEM, et al. A brief cognitive‐behavioral intervention for treating depression and panic disorder in patients with noncardiac chest pain: A 24‐week randomized controlled trial. Depression and anxiety. 2013;30(7):670-8.

Wuthrich VM, Rapee RM. Telephone-Delivered Cognitive Behavioural Therapy for Treating Symptoms of Anxiety and Depression in Parkinson's Disease: A Pilot Trial. Clin Gerontol. 2019 Jul-Sep;42(4):444-453. doi: 10.1080/07317115.2019.1580811.

Yorke J, Adair P, Doyle AM, Dubrow-Marshall L, Fleming S, Holmes L, et al. A randomised controlled feasibility trial of Group Cognitive Behavioural Therapy for people with severe asthma. Journal of Asthma. 2016:0.

Appendix C. Funnel plot


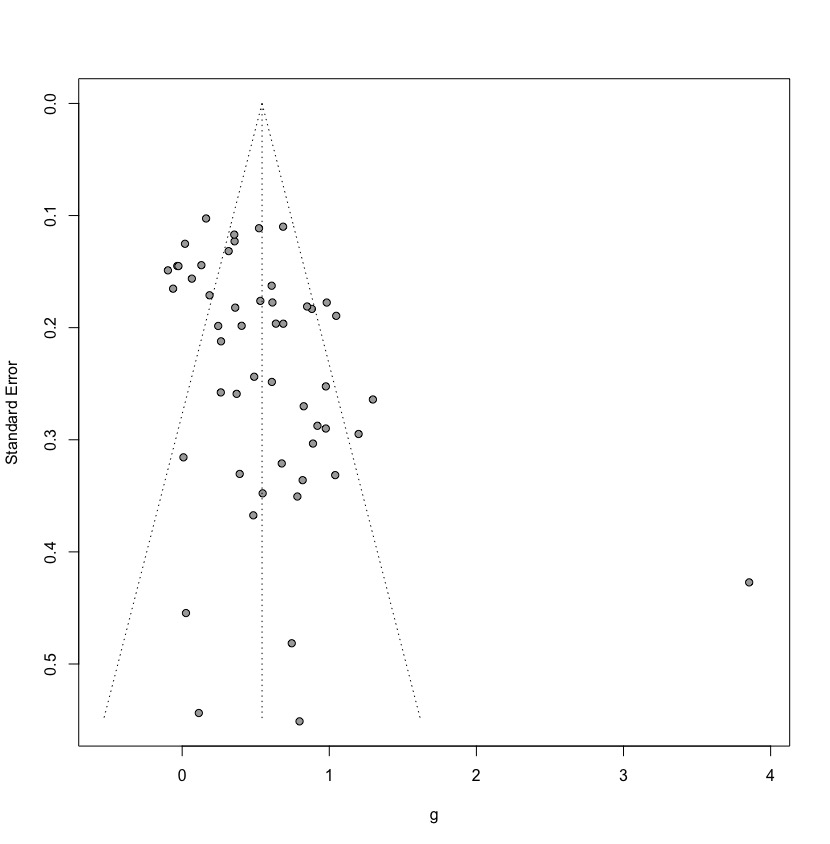


Appendix D. Outcomes at 6 months post-randomisation

| ***Outcomes*** | ***k*** | ***g*** | **CI** | ***I*^2^** | **CI** | **Prediction Interval** | **NNT** |
| --- | --- | --- | --- | --- | --- | --- | --- |
| Depression and anxiety combined ^a)^ |  |  |  |  |  |  |  |
| All comparisons (effect sizes combined) | 12 | 0.52 | 0.21; 0.83 | 86 | 78; 91 | -0.51; 1.55 | 6.13 |
| All studies (effect sizes combined) | 10 | 0.43 | 0.11; 0.76 | 87 | 77; 92 | -0.56; 1.43 | 7.61 |
| Three-Level Model (CHE) ^b)^ | 28 | 0.53 | 0.22; 0.84 | 85 | - | -0.44; 1.50 | 6.03 |
| One effect size per study (lowest) | 10 | 0.32 | 0.02; 0.61 | 81 | 65; 89 | -0.54; 1.17 | 10.90 |
| One effect size per study (highest) | 10 | 0.54 | 0.15; 0.94 | 86 | 76; 92 | -0.65; 1.73 | 5.88 |
| Outliers removed^c)^ | 10 | 0.65 | 0.35; 0.95 | 78 | 60; 88 | -0.24; 1.54 | 4.74 |
| Adjusted for publication bias | 16 | 0.28 | -0.07; 0.63 | 88 | 81; 92 | -1.04; 1.61 | 12.48 |
| Only rob > 3 | 5 | 0.20 | -0.08; 0.49 | 66 | 11; 87 | -0.47; 0.88 | 17.69 |
| All effect sizes (assuming independence) | 28 | 0.53 | 0.35; 0.71 | 79 | 69; 85 | -0.29; 1.35 | 6.03 |
| Depression only ^a)^ |  |  |  |  |  |  |  |
| All comparisons (effect sizes combined) | 11 | 0.63 | 0.31; 0.94 | 79 | 64; 88 | -0.33; 1.58 | 4.97 |
| All studies (effect sizes combined) | 9 | 0.55 | 0.21; 0.88 | 81 | 64; 90 | -0.4; 1.50 | 5.82 |
| Three-Level Model (CHE) ^b)^ | 14 | 0.63 | 0.32; 0.94 | 80 | - | -0.29; 1.54 | 4.95 |
| One effect size per study (lowest) | 9 | 0.49 | 0.17; 0.81 | 78 | 59; 89 | -0.39; 1.37 | 6.59 |
| One effect size per study (highest) | 9 | 0.59 | 0.22; 0.95 | 81 | 64; 90 | -0.43; 1.60 | 5.36 |
| Outliers removed^d)^ | 10 | 0.69 | 0.37; 10 | 74 | 51; 86 | -0.21; 1.59 | 4.43 |
| Adjusted for publication bias | 14 | 0.44 | 0.08; 0.80 | 82 | 71; 89 | -0.79; 1.66 | 7.49 |
| Only rob > 3 | 5 | 0.26 | -0.05; 0.58 | 62 | 0; 86 | -0.46; 0.99 | 13.28 |
| All effect sizes (assuming independence) | 14 | 0.62 | 0.36; 0.88 | 75 | 59; 85 | -0.24; 1.48 | 4.99 |
| Anxiety only ^a)^ |  |  |  |  |  |  |  |
| All comparisons (effect sizes combined) | 11 | 0.54 | 0.21; 0.87 | 81 | 66; 89 | -0.47; 1.55 | 5.91 |
| All studies (effect sizes combined) | 9 | 0.46 | 0.11; 0.8 | 82 | 66; 90 | -0.53; 1.44 | 7.20 |
| Three-Level Model (CHE) ^b)^ | 13 | 0.54 | 0.21; 0.87 | 83 | - | -0.44; 1.52 | 5.91 |
| One effect size per study (lowest) | 9 | 0.40 | 0.09; 0.71 | 79 | 60; 89 | -0.47; 1.27 | 8.32 |
| One effect size per study (highest) | 9 | 0.49 | 0.1; 0.88 | 83 | 68; 90 | -0.61; 1.59 | 6.60 |
| Outliers removed^d)^ | 10 | 0.61 | 0.29; 0.93 | 73 | 50; 86 | -0.30; 1.52 | 5.13 |
| Adjusted for publication bias | 15 | 0.29 | -0.08; 0.67 | 83 | 73; 89 | -1.03; 1.61 | 12.01 |
| Only rob > 3 | 5 | 0.14 | -0.14; 0.43 | 56 | 0; 84 | -0.49; 0.78 | 26.09 |
| All effect sizes (assuming independence) | 13 | 0.49 | 0.22; 0.76 | 77 | 60; 86] | -0.36; 1.34 | 6.60 |

a) In the analyses of influential cases no studies were removed, so the results are identical to the main analyses

b) The results of the multilevel model, using robust variance estimation, were alsmost identical to those of the three-level CHE model. Therefore we only report the results of the CHE model.

c) Excluded studies: Lam, 2010, Patel, 2003

d) Excluded study: Lam, 2010

Appendix E. Outcomes at 12 months post-randomisation

| ***Outcomes*** | ***k*** | ***g*** | **CI** | ***I*^2^** | **CI** | **Prediction Interval** | **NNT** |
| --- | --- | --- | --- | --- | --- | --- | --- |
| Depression and anxiety combined ^a)^ |  |  |  |  |  |  |  |
| All comparisons (effect sizes combined) | 6 | 0.00 | -0.24; 0.24 | 41 | 0; 77 | -0.35; 0.35 | >100 |
| Three-Level Model (CHE) ^b)^ | 13 | 0.01 | -0.21; 0.22 | 28 | - | -0.30; 0.31 | >100 |
| One effect size per study (lowest) | 6 | -0.11 | -0.35; 0.12 | 35 | 0; 74 | -0.32; 0.09 | 37.13 |
| One effect size per study (highest) | 6 | 0.05 | -0.2; 0.31 | 34 | 0; 73 | -0.37; 0.47 | 72.76 |
| Influence Analysis^c)^ | 3 | 0.03 | -0.01; 0.06 | 0 | 0; 90 | -1.32; 1.37 | >100 |
| Adjusted for publication bias | 9 | -0.09 | -0.35; 0.19 | 56 | 8; 79 | -0.72; 0.55 | ^d)^ |
| Only rob > 3 | 2 | -0.07 | -0.96; 0.82 | 0 | ^e)^ | ^e)^ | 59.26 |
| All effect sizes (assuming independence) | 13 | 0.01 | -0.13; 0.16 | 32 | 0; 65 | -0.26; 0.29 | >100 |

a) There were no multi-arm trials therefore, the pooled effect sizes across trials and comparisons were identical. In the outlier analyses, no outliers were detected, so the outcomes were identical to the main analyses.

b) The results of the multilevel model, using robust variance estimation, were alsmost identical to those of the three-level CHE model. Therefore we only report the results of the CHE model.

c) Removed as influential cases: Lam, 2010; Patel, 2003; Scheidt, 2013.

d) The NNT cannot be calculated when the effect size is negative.

e) the 95% CI of I2 and the prediction interval cannot be calculated when the number of studies is smaller than 3.
